# Fitness Matters as We Age: A Celebration of the VA Gerofit Program

**DOI:** 10.1093/geroni/igaa057.2785

**Published:** 2020-12-16

**Authors:** Miriam Morey, Cathy Lee

## Abstract

In recognition of the GSA’s 75th Anniversary “Why Age Matters” we celebrate the 7th anniversary of the Gerofit dissemination initiative. Gerofit is an exercise and health promotion program for older Veterans that has been declared a Veterans Health Administration (VA) “Best Practice” and been disseminated to 17 VA’s across the country. Over 7000 Veterans have participated in Gerofit initiated programs and have reported robust outcomes including improved quality of life, physical and mental health, and high levels of satisfaction with the programs. For this symposium, we focus on newly acquired program outcomes that emphasize the importance of fitness as we age. The first paper compares hospitalization and emergency room visits between individuals participating in Gerofit for 12 months compared to a matched control group. The second paper describes four-year trajectories of physical performance to highlight the impact of becoming fit over expected normative trajectories. The third paper examines outcomes of a home-based geriatric walking clinic. The fourth paper describes the impact of exercise adherence on chronic pain. The fifth paper describes changes in medication utilization compared to a matched control group following 12-months of supervised exercise. These papers highlight the importance of fitness as a contributor to overall health during the aging process and celebrates that fitness matters, no matter when you start!

